# United in Diversity, Divided in Adversity? Support for Right-Wing Eurosceptic Parties in the Face of Threat Differs Across Nations

**DOI:** 10.3389/fpsyg.2019.01880

**Published:** 2019-08-14

**Authors:** Matteo Vergani, Ana-Maria Bliuc, Avelie Stuart, Constantina Badea, Daniela Muntele, Craig McGarty

**Affiliations:** ^1^Alfred Deakin Institute for Citizenship and Globalisation, Geelong, VIC, Australia; ^2^School of Social Sciences and Psychology, University of Dundee, Dundee, United Kingdom; ^3^Department of Psychology, University of Exeter, Exeter, United Kingdom; ^4^UFR Sciences Psychologiques et Sciences de l’Education (SPSE), Université Paris Nanterre, Nanterre, France; ^5^School of Psychology, Alexandru Ioan Cuza University, Iaşi, Romania; ^6^School of Social Sciences and Psychology, Western Sydney University, Sydney, NSW, Australia

**Keywords:** intergroup attitudes, threat, political support, Euroscepticism, far-right

## Abstract

This article investigates whether the perceived threat of terrorism explains the support for right-wing Eurosceptic parties and Euroscepticism above and beyond other relevant variables, including perceived economic and immigration threats. We first examined the entire Eurobarometer samples of 2014 and 2015, and then conducted survey experiments in four European Union (EU) countries, that is, United Kingdom (*N* = 197), France (*N* = 164), Italy (*N* = 312), and Romania (*N* = 144). Our findings suggest that the perceived threat of terrorism has a small effect on the negative attitudes toward the EU above and beyond the effect of immigration and economic threats and other basic control variables. The relationship between these variables varies across countries and it is less linear than we might expect.

## Introduction

Euroscepticism is a loosely defined transnational socio-political movement, often associated with populist political parties, that implies rejection of the European integration project and the opposition to one’s country joining or remaining a member of the European Union (EU, Taggart and Szczerbiak, 2004). Especially after the so-called Brexit referendum of June 24, 2016, identifying the specific drivers of Euroscepticism has become increasingly important for both academics and political analysts. For the EU states and its citizens, Euroscepticism threatens to undermine the unity of the EU at a time when Europe faces complex challenges, including the Brexit process, terrorist threats, military conflicts in bordering regions, economic and financial adjustments, and changing immigration flows. These challenges may themselves increase support for Eurosceptic parties that threaten the existence of the European Union as we know it today.

In this article, we examine the role of the threat of terrorism in the context of other threats. Based on media reports and emerging research, it is plausible that the threat of terrorism may have played a role in increasing the electoral support for Euroscepticism and right-wing Eurosceptic parties: for example, the results of the 2015 French regional elections might suggest that electoral support for the right-wing Eurosceptic National Front increased in the wake of the Paris terrorist attacks (Viette and Todd, 2015). However, rigorous research looking at European attitudes before and after the Paris terrorist attacks shows that there is no evidence of average impacts across a range of issues, from xenophobia, political attitudes and policy preferences (Castanho Silva, 2018). Similarly, a subsequent study found no evidence of the impact of the 2016 Berlin terrorist attack on anti-immigration, anti-refugee and anti-European Union sentiment (Larsen et al., 2019).

There is extensive research on the factors that can help explain negative views of the EU, such as those associated with right-wing Euroscepticism; these factors include socio-demographic individual characteristics and political orientation (Gabel and Palmer, 1995), national pride (Carey, 2002), and economic expectations (Serricchio et al., 2013). Negative attitudes toward the EU were also found to be associated with religious intolerance and anti-Muslim sentiment (Hobolt et al., 2011), anti-immigration attitudes, and perceived immigrant threat (Riek et al., 2006).

Building on research on the effect of various threats on intergroup relations, the aim of this article is to investigate the relationships between perceived threats (i.e., terrorism, but also economic and immigration) and support for Euroscepticism and right-wing Eurosceptic parties. Our study focuses on 2014 and 2015, which are the years immediately before the build-up to the Brexit referendum that changed the perception and meaning of Euroscepticism in the whole European Union. As a background to our research, we start by discussing the effect of different types of threats on Euroscepticism, then argue that so far the effect of terrorism threats has not been given the attention it deserves in the literature on Euroscepticism.

### Perceived Threat and Political Attitudes

Previous research found a stable significant association between perceived threat, particularly immigration threat, and political attitudes. Surveys collected in the United Kingdom after the Brexit referendum suggest that there is a direct association between anti-immigration and anti-EU views, especially among low-income and unemployed voters, those living in low-skilled areas, and with lower education levels (Arnorsson and Zoega, 2016; Ashcroft and Bevir, 2016; Wright et al., 2016). Perceived immigration threat was found to predict voting for right-wing Eurosceptic parties over and above other factors, such as individual characteristics and socio-economic contexts (Werts et al., 2012). This is not surprising, because in their rhetoric, such parties do not only seek to distance their country from the European project, but also argue for restrictive immigration policies, and usually express prejudice toward immigrants (Treib, 2014). Prior research has consistently found associations between policy attitudes such as support for exclusionary policies, expulsion of immigrants from the EU, and perceived threats of immigration and economic downfall (McLaren, 2003). More generally, McLaren and Johnson (2007) found that economic and immigration threats have predicted anti-immigration hostility in the United Kingdom, and Pereira et al. (2009) found that perceptions of threat (especially realistic threat, i.e., related to crime and economic hardships) mediated the relationship between prejudice and discrimination of immigrants in Europe.

Anti-immigration attitudes and intergroup processes (including attitudes toward outgroups), drive the association between different forms of perceived threat and political preferences (such as support for anti-immigration policies and right-wing political parties). Drawing on integrated threat theory (ITT), Stephan et al. (2000) found that an integrated system of four threats predicts negative attitudes toward outgroups: realistic threats, symbolic threats, intergroup anxiety and negative stereotypes. The theory was revised in 2002 synthetizing the four components into two basic types: symbolic and realistic threats (Stephan and Renfro, 2002). In general, a symbolic threat captures the perception that an out-group has different cultural worldviews than the in-group (Sears and Kinder, 1981) and a realistic (or material) threat refers to the threat that an out-group poses to in-group resources (McLaren and Johnson, 2007). While these types of threats can be experimentally distinguished, in the real world, immigration and economic hardships are often interlinked and encompass multiple dimensions. That is, perceived immigration threat usually encompasses realistic and symbolic threats, as well as a threat to personal safety when people perceive immigration to be associated with increased crime (McLaren and Johnson, 2007).

Despite this relatively large body of research on factors associated with Euroscepticism, the literature has so far failed to take into account, in the context of other existing perceived threats, the effect of a potentially highly significant perceived threat that has become prominent in Europe: the perceived threat of terrorism. We test here whether the *perceived terrorism threat* is a distinct predictor of Euroscepticism above and beyond other perceived threats. We next describe existing research that supports this proposition.

### Perceived Terrorism Threat

Research from the United States shows that perceived terrorism threat is associated with hawkish political attitudes (such as support for restrictive domestic policies or aggressive foreign policies) across partisan lines, even when taking into account individual characteristics, such as religious rites and attendance at places of worship (Huddy et al., 2005; Gadarian, 2010, 2014). For example, Gadarian (2014) found that exposure to terrorism imagery enhanced candidate evaluations and increased approval for President Bush even among Democrats. Huddy et al. (2005) found that terrorism threat perception increases support for surveillance of Arab Americans, security checks for Arab visitors and greater restrictions on visas. Although sometimes terrorism is linked to religious conflict in popular media and public discourse (especially in the current context of the high perceived threat from terrorist groups such as al-Qaeda and ISIL, Islamic State of Iraq and the Levant), these findings suggest that terrorism threat does not exclusively trigger anti-religious minority group sentiment and out-group aggression in the form of religious intolerance, but also influences more general political attitudes such as policy preferences and support for political leaders. Thus, perceived terrorism threat appears to be a distinct concept that goes beyond political and religious orientations. Perceived terrorism threat was also found to be associated with punitive and aggressive worldviews, ethnocentrism, and rally effects [i.e., a short term increase in popular support for the President in times of crisis, Hetherington and Nelson (2003), Ladd (2007)].

In the European context, there is abundant cross-sectional evidence about the relationship between perceived terrorism threat and negative views of immigrants (e.g., Uenal, 2016), but more contradicting experimental evidence. For example, a quasi-experimental study conducted in Spain before and after the terrorist attacks in Madrid (March 11, 2004) provided findings consistent with the research conducted in the United States, that is, the attacks increased prejudice against out-groups (i.e., Arabs and Jews in this study), authoritarianism, and attachment to reactionary and conservative values (Echebarria-Echabe and Emilia, 2006). Conversely, two studies measuring attitudes before and after the Paris 2015 attacks (Castanho Silva, 2018) and the 2016 Berlin attacks (Larsen et al., 2019) found no effects of terrorist attacks on attitudes to migrants, policy preferences on migration, xenophobia, and other political attitudes. An experimental study conducted in Italy found that increased perceived terrorism threat from ISIL, triggered through reading a newspaper article about the terrorist group’s threats to the Vatican, makes Italian Catholics more supportive of conservative political leaders, and more supportive of politicians stating that in Italy “there is no space for Mosques” (Vergani and Tacchi, 2016, p. 1894). Four experimental studies conducted in Germany found that terrorism salience leads to increased system-justification, with a medium average effect size (*d* = 0.47) (Ullrich and Cohrs, 2007). Yet, no research has looked at the relationship between perceived terrorism threat and political attitudes in the context of other factors such as immigration and economic threats. We expect that perceived terrorism threat will be strongly associated with material (economic) and symbolic (immigration) threats, especially in the European political context. In this article, we empirically investigate this relationship.

We acknowledge that terrorism is a multidimensional source of perceived threats that can also be associated with both symbolic threats and material concerns (Oswald, 2005). We propose that terrorism encompasses two distinctive dimensions, namely *personal safety threat* and *existential threat*. The evidence for a *personal safety threat* dimension is evident in the findings that personal safety issues, which were consistently associated with anti-Arab attitudes, were a significant concern for participants following the 9/11 attacks (Oswald, 2005). The *existential threat* dimension is derived from terror management theory, where research shows that any reminders of terrorism heighten existential concerns (Pyszczynski et al., 2003), that in turn activate a striving for self-esteem and the need for world defense in the form of increased support for conservative and security-oriented political leaders (Landau et al., 2004), and support for extreme military force and restrictive domestic policies (Pyszczynski et al., 2006). Drawing on these findings, we anticipate that perceived terrorism threat is likely to contribute to models predicting Euroscepticism, over and above other types of threat and other relevant predictors. We tested this proposition in two studies, as follows.

## Study 1

To examine the relationship between perceived terrorism threat and Euroscepticism that includes other types of threat and predictors, we analyzed data collected by two Eurobarometer surveys: Eurobarometer 81.4 (May–June 2014) and Eurobarometer 83.3 (May 2015). The two surveys samples are residents of each EU member state aged 15 years and over, with a sample size of 26,540 (2014) and 26,214 (2015). The survey questionnaires contain questions that enable the construction of proxy measures relating to the perceived threat of terrorism, immigration and economic hardship, as well as negative attitudes toward the EU.

### Measures

We created comparable measures of perceived terrorism, immigration and economic threats by combining the answers to the following questions: “What do you think are the two most important issues facing (OUR COUNTRY) at the moment?” or “What do you think are the two most important issues facing our Community at the moment?”; “Personally, what are the two most important issues you are facing at the moment?”; “What do you think are the two most important issues facing the EU at the moment?”

To answer these questions, participants could select two answers from a list of 13 options, among which are “terrorism,” “immigration,” and “economic situation.” We created three different measures by recording the responses to these questions:

(1) An index of *perceived terrorism threat*, obtained by adding each participant’s selection of the choice “terrorism” in any of the answers to the questions above (0 mentions = 0 of “terrorism” in the answers to the questions above, 1 mention = 1; 2 mentions = 2; 3 mentions = 3);
(2) An index of *perceived immigration threat*, obtained by adding each participant’s selection of the choice “immigration” (see above); and
(3) An index of *perceived economic threat*, obtained by adding each participant’s selection of the choice “economic situation” (see above).

Each measure has four different levels, corresponding to zero, one, two, and three instances where the words “terrorism,” “immigration,” and “economic situation” are selected as answers to the questions above.

To capture *negative attitudes toward the EU*, we used the question: “What does the EU mean to you personally?” To answer this question, participants could select from a list of 14 possible answers, among which are: “Unemployment,” “Waste of money,” “Loss of our cultural identity,” “More crime,” and “Not enough control at external borders.” We created the measure by assigning one point to each instance where any of those negative statements were selected, and zero for no instances, and then we combined the answers by adding the scores. The measure had six levels corresponding to zero, one, two, three, four, and five instances where negative connotations attributed to the EU were selected.

We expect perceived terrorism threat to be linked to respondents’ agreement with those negative statements attributed to the EU because terrorism threat encompasses a threat to personal safety (related to the fear of crime and support for stricter border control), a symbolic threat (related to the loss of cultural identity), and a material threat (related to unemployment and loss of resources). We also expect that there will be overlaps between the measures of terrorism threat, immigration and economic threat, because they all tap into both symbolic and material threats.

We also extracted basic demographic and ideological variables to be used as controls, specifically age, gender and political ideology (i.e., “In political matters people talk of the left and the right. How would you place your views on this scale?” Answers range from 1 = “left” to 10 = “right”).

### Results

To test whether terrorism threat contributes to models predicting negative attitudes toward the EU over and above other types of threat and other relevant predictors, we conducted both bivariate correlations between the main variables of interest (negative attitudes to the EU and perceived economic, immigration and terrorism threats) and hierarchical OLS regressions. In the regression models in the first block we included the control variables (gender, age and political ideology); in the second step the proxy measures of economic and immigration threats, and in the third step the measure of perceived terrorism threat (Table 1).

**TABLE 1:** Standardized regression coefficients on negative attitudes to the EU (higher values mean higher negative associations with the EU).

	**2014**	**2015**
Age	0.13^∗∗∗^	0.14^∗∗∗^
Gender (female)	–0.02^∗∗^	–0.01
Political ideology	–0.00	0.00
Block 1 Adjusted R^2^	0.02	0.02
Age	0.12^∗∗∗^	0.13^∗∗∗^
Gender (female)	–0.02^∗∗^	–0.01
Political ideology	–0.01	–0.00
Economic threat	–0.01	0.00
Immigration threat	0.12^∗∗∗^	0.10^∗∗∗^
Block 2 Adjusted R^2^	0.03	0.03
Age	0.12^∗∗∗^	0.13^∗∗∗^
Gender (female)	–0.02^∗∗^	–0.01
Political ideology	–0.01	–0.00
Economic threat	–0.01	0.00
Immigration threat	0.13^∗∗∗^	0.10^∗∗∗^
Terrorism threat	0.02^∗∗∗^	0.02^∗∗∗^
Block 3 Adjusted R^2^	0.03	0.03
Block 3 F-value	149.844	130.718
Sample size	26540	26214

*^∗∗^*p* < 0.01, ^∗∗∗^*p* < 0.001.*

The bivariate correlations show that, in the 2014 sample, negative attitudes to the EU are significantly associated with economic threat (*r* = −0.02, *p* < 0.00), immigration threat (*r* = 0.13, *p* < 0.00) and terrorism threat (*r* = 0.02, *p* < 0.00). In 2015, negative attitudes to the EU are significantly associated with immigration threat (*r* = 0.11, *p* < 0.00 and terrorism threat (*r* = 0.03, *p* < 0.00), but not with economic threat (*r* = −0.01, *p* = 0.06).

As illustrated in Table 1, perceived terrorism threat does add variance above and beyond immigration and economic threat. The variance explained is greater than political ideology or economic threat, yet the size of the effect is small.

**FIGURE 1:**
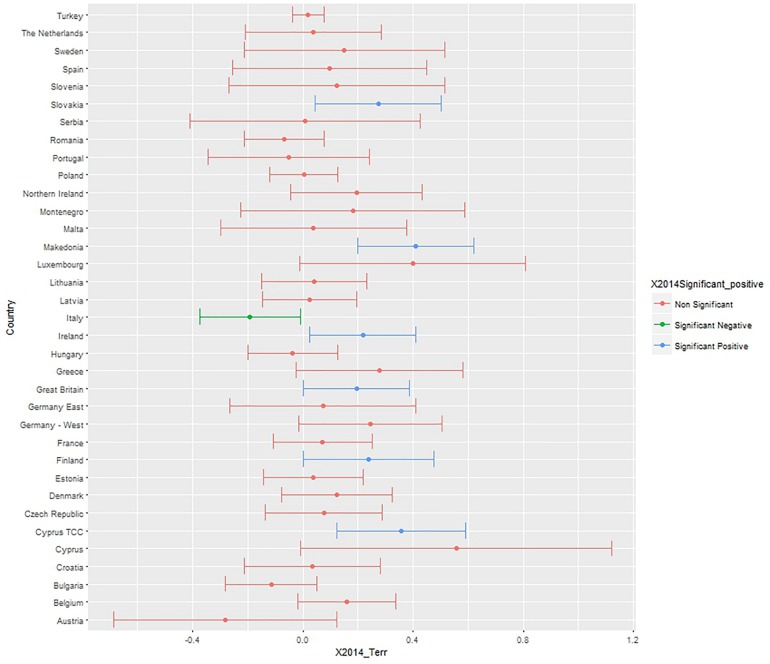
Unstandardized B and confidence intervals of the terrorism threat coefficient predicting negative attitudes toward the EU, controlling for age, gender, political orientation, realistic, and symbolic threats. Data: Eurobarometer 2014.

**FIGURE 2:**
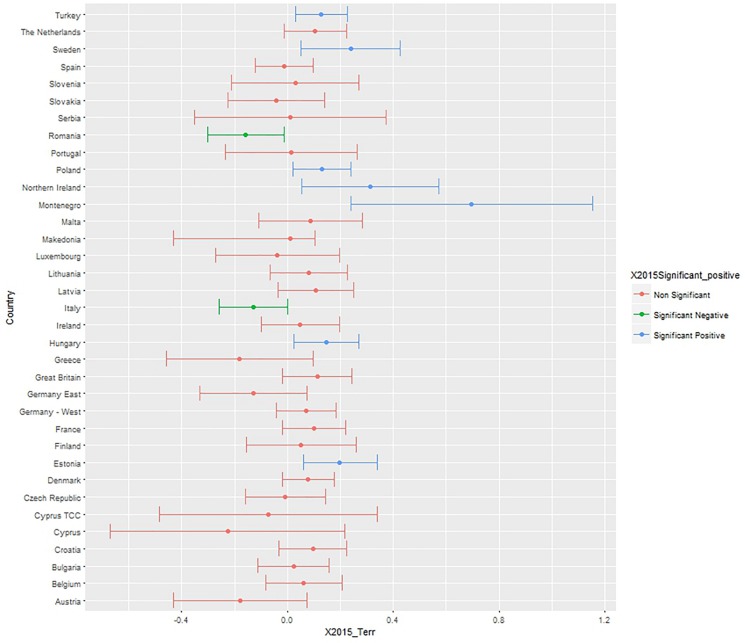
Unstandardized B and confidence intervals of the terrorism threat coefficient (*x*-axis) predicting negative attitudes toward the EU, controlling for age, gender, political orientation, realistic, and symbolic threats. Data: Eurobarometer 2015. Red bars indicate non-significant associations, blue significant and positive associations, and green significant and negative associations.

To test whether the perceived threat of terrorism consistently predicts negative attitudes toward the EU at country levels, we conducted OLS regressions for each country sample, and we plotted the unstandardized coefficient and confidence intervals (*x*-axis), highlighting the significant coefficients with green for significant negative, and blue for significant positive predictors (Figures 1, 2). Red bars indicate non-significant associations, blue significant and positive associations, and green significant and negative associations.

In 2014, more negative attitudes toward the EU were significantly associated with higher terrorism threat perception in Slovakia, Macedonia, Ireland, Great Britain, Finland, and Cyprus TCC, whereas in Italy less negative attitudes to the EU were associated with higher terrorism threat. It is worth noting that the confidence intervals are large in almost all countries, and the relationship between perceived terrorism threat and attitudes toward the EU is not statistically significant for most countries.

In 2015, more negative attitudes to the EU were significantly associated with higher perceived terrorism threat in Turkey, Sweden, Poland, Montenegro, Hungary, and Estonia, whereas in Romania and Italy there was a statistically significant association between less negative attitudes to the EU and higher perceived terrorism threat. Also in 2015, the relationship between terrorism threat and attitudes to the EU was not statistically significant for most countries.

Perceived terrorism threat was a significant predictor of negative attitudes toward the EU over and above other types of threat and other relevant predictors in six countries in 2014 (Slovakia, Macedonia, Ireland, Great Britain, Finland, and Cyprus) and seven countries in 2015 (Turkey, Sweden, Poland, United Kingdom, Montenegro, Hungary, Estonia). Remarkably, perceived terrorism threat was associated with positive attitudes to the EU in Italy (in 2014 and 2015) and in Romania (in 2015).

### Discussion

The regression models on the whole Eurobarometer sample show that the perceived threat of terrorism has a small positive effect on the negative attitudes toward the EU above and beyond the effect of immigration and economic threats and other basic control variables. The reason why the effect is small could be due to key contextual differences across countries. Although the relationships between perceived terrorism threat and negative attitudes toward the EU change to some degree from year to year, and the confidence intervals can be large (making it unreliable to make claim about specific country level differences) – we note an increasing trend toward a higher number of countries being Eurosceptic. Nonetheless, there are also countries like Italy and Romania where being pro-EU is associated with a higher terrorism threat. Data from the most recent Eurobarometer (85.2, May 2016), indicates that significant diversity in the relationship between perceived terrorism threat and negative views of the EU across countries has been sustained (see xsSupplementary Materials for the analysis of the 2016 Eurobarometer data).

In Study 1, the proxy measures of perceived threat can be seen as relatively imprecise and do not allow for further investigation of the country differences and the relationships with other relevant predictors of Euroscepticism. A higher number of instances where negative associations with the EU are mentioned do not equate to how strongly participants feel about each of these negative attitudes, i.e., the measure of negative attitudes toward the EU and any perceived threat do not capture the strength of the participants’ attitudes. Moreover, as participants cannot indicate more than two types of threat per category, it is impossible to have high values on all types of threat. This on the one hand limits respondents to indicate to perceive threat from multiple sources, on the other hand forces respondents to rank threats in order to identify the most salient ones. As the Eurobarometer does not provide any other proxy measure of perceived threat, we decided to investigate country differences in a second study, where we collected data using more accurate measures of perceived threat and more accurate predictors of Euroscepticism. We decided to incorporate experimental manipulations into our survey (which will be described in the next section), to test whether we could establish a causal link between perceived terrorism threat and negative attitudes toward the EU. Additionally, we wanted to measure Euroscepticism in terms of both support for Eurosceptic parties and support for Eurosceptic policies (with no mention of political parties), in order to have a clearer understanding of the relationship between perceived terrorism threat and political attitudes toward the EU versus national political parties.

## Study 2

To ensure a broad representation of the different relationships between perceived terrorism threat and negative attitudes toward the EU, as emerged from Study 1, we collected data from four EU countries: United Kingdom (*N* = 197), France (*N* = 164), Italy (*N* = 312), and Romania (*N* = 144). In Study 1, we found that the relationship between terrorism threat and negative attitudes toward the EU were either negative or non-significant in Italy and Romania, or either positive or non-significant in Great Britain and France. Moreover, by including data from both Western and Eastern European countries, we expected to include a variety of contexts that well represent the diversity that emerged from Study 1.

According to the Standard Eurobarometer 83, which presented data collected in May 2015 (from the official data collection), Romanian citizens’ trust in the European Union was 68%, in Italy it was 36%, 32% in France and 29% in the United Kingdom. This data highlights the crisis the EU is facing in terms of public opinion in two of the six founding member states, Italy and France, and the United Kingdom is one of seven nations that joined the Union in 1973. Diminished trust in the EU has been associated with the economic crisis in Europe and the rise of Eurosceptic parties such as the Lega Nord in Italy, the UKIP in the United Kingdom and the Front National in France. The Romanian context was different because the country joined the EU in 2007 after a long transition, and the EU has been viewed by the public there as a way out of the hardships associated with the socialist past (Papadimitriou and Phinnemore, 2008). Despite the different context and historical relations with the EU, Romania has also seen the rise of far-right parties such as Romania Mare, the main nationalist and Eurosceptic populist party in the country.

We conducted Study 2 to examine the nature and direction of the relationship between the perceived threat of terrorism, Euroscepticism, and support for Eurosceptic parties in the different contexts represented by Italy, France, United Kingdom, and Romania.

### Procedure

We collected web-based and paper-based questionnaires from participants at universities in four countries in their respective languages (English, French, Italian, and Romanian) between February and June 2015. Written and informed online consent was obtained from the study participants in Italy, United Kingdom, and France. In Romania, where there is no ethics process in place in universities for non-medical research which involves human participants, our consent process complied with the ethical standards of the university of the lead researcher: the questionnaires were completely anonymous (no identifying information was collected), and the participation was completely voluntary (and participants were provided with a plain language statement and made aware of opt-out consent before starting the survey). To protect the complete anonymity of participants and ensure their privacy, only opt-out consent was obtained from participants. The informed consent of the participants was implied through survey completion.

After collecting demographic information and measures of political orientation and national identification, we randomly assigned participants to one control condition and two experimental conditions where we manipulated the salience of terrorism threat and respectively of economic threat:

(1) *Terrorism threat condition* – participants were asked to answer two open-ended questions: “What sort of things do you think about when you think about the Islamic State? (The Islamic State is the rebel group that currently controls territory in Iraq and Syria)” and “How does the thought of a possible terrorist attack in [participant country] from the Islamic State make you feel?”
(2) *Economic threat condition* – participants were asked to answer two open-ended questions: “What sort of things do you think about when you think about the economic crisis?” and “How does the thought of future worsening of the economic crisis in [participant country] make you feel?”
(3) *No threat (control) condition* – participants were not asked any questions.

Following the random allocation of participants to the experimental conditions, they were asked to complete, as manipulation checks measures of perceived threat (from immigration, economic crisis and terrorism) and then measures capturing the dependent variables (i.e., measures of support for the EU and for Eurosceptic parties). We included the measure of perceived immigration threat for consistency with Study 1, and to control in the analysis whether the perceived threat of terrorism affects Euroscepticism over and beyond the effects of the perceived threat from immigrants.

### Materials

We included questions for gender, age and their parents’ education. We did not include a question about the education of the respondents because, as we collected questionnaires among university students, we did not expect to obtain enough variability in education. Specifically, we asked participants to identify the highest level of education of each of their parents (1 = primary, 2 = secondary, 3 = tertiary education), and we computed a combined measure of the two scores.

To measure *political orientation* and *national identity*, we included in our questionnaire a political orientation measure (a scale from left-wing = 1 to right-wing = 10). Additionally, we asked participants to rate on a scale of 1–4 their agreement with the statement “Would you say you are proud to be from [participant country]?” Previous research has suggested that political orientation and national identity predict Euroscepticism (Gabel and Palmer, 1995; Carey, 2002).

To capture *identification with the EU*, we used a 10 item scale adapted from the group-level self-investment scale developed by Leach et al. (2008): specifically, we included the solidarity subscale (e.g., “I feel a bond with other Europeans”), the satisfaction subscale (e.g., “I think that Europeans have a lot to be proud of”) and the centrality subscale (e.g., “Being European is an important part of how I see myself”).

To capture *perceived threat*, we adapted seven items to assess realistic and terrorism threat from Huddy et al. (2005).^1^ Additionally, we collected a measure of perceived threat from immigrants, to test whether the perceived threat of terrorism affects views of Euroscepticism over and above this variable. Following previous research that used views of immigrants and ethnic minorities as a proxy of symbolic threat (McLaren, 2003; Lubbers and Jaspers, 2011), we asked participants to rate their agreement on a scale of 1–4 with the following statements: “Immigrants contribute a lot to our community,” “Legally established immigrants from outside [participant country] should be able to become naturalized easily,” and “The right to asylum in [participant country] should be easier to obtain.”

To measure *support for Eurosceptic parties*, we asked participants to rate on a scale of 1–4 their support for Eurosceptic parties through the following item: “I support (Eurosceptic party, e.g., [UKIP/Front National/Lega Nord/România Mare] because it defends the people in [participant country] from the invasion of immigrants” and “I support e.g., [UKIP/Front National/Lega Nord/România Mare] because it wants to exit the European Union.”

To measure *negative attitudes toward the EU*, we collected participants’ agreement with one item that captured negative attitudes toward the EU without mentioning any Eurosceptic party: “Taking everything into consideration, people from [participant country] would benefit from the exit of ([participant country] from the European Union.” We consider this stimulus to be an accurate measure of Euroscepticism, defined at its core as opposition to the EU (Taggart and Szczerbiak, 2004). We decided to use this measure because we wanted to capture opposition to the EU without politicizing the issue of Euroscepticism using the name of a specific party, which could have created an ideological rejection of the stimuli. See the Supplementary Materials for the reports of the internal consistency of the combined measures described above.

### Results

Table 2 shows that there were differences between the samples in terms of demographic characteristics. Participants in the United Kingdom and Italy were slightly younger than participants in France and Romania; however, we did not find any significant correlation between age and other variables, therefore we did not include age in the subsequent analyses (see correlation tables in the Supplementary Materials). We also had fewer women participants in the French sample (60.4% compared to about 80% of females in the other country samples), and participants’ parents were significantly more educated in the United Kingdom and France compared to Italy and Romania. These gender and education differences could be important in explaining the sample’s support for Euroscepticism, and therefore we accounted for them in the subsequent analyses.

**TABLE 2:** Demographic characteristics of the samples.

	**United Kingdom (*N* = 197)**	**France (*N* = 164)**	**Italy (*N* = 312)**	**Romania (*N* = 144)**
Age (mean, SD)	20.88 (3.30)	21.05 (2.85)	20.54 (3.58)	22.72 (2.07)
Females (%)	82.0	60.4	81.1	84.5
Parents’ education^∗^	2.36 (0.39)	2.35 (0.66)	1.92 (0.64)	1.98 (0.56)

^*^Primary = 1, secondary = 2, tertiary = 3.

Next, we conducted one-way ANOVA tests to assess whether the manipulations affected threat perception and, consequently, views of Euroscepticism. As we tested multiple dependent variables (i.e., three threat perceptions and two measures of Euroscepticism) we reduced familywise error rate adopting Bonferroni correction to reduce the probability of incurring type I errors. The ANOVA tests yielded no significant differences between groups in the experimental and control conditions in the United Kingdom (all *p*-values above 0.15), Italy (all *p*-values above 0.36), and in France (all *p*-values were above 0.52), except for the dependent variable capturing views of the EU, for which participants in the terrorism condition (*M* = 1.83, SD = 0.71) reported more negative views of the EU compared to participants in the crisis (*M* = 2.05, SD = 0.81) and control (*M* = 2.23, SD = 0.82) conditions, *F*(2, 161) = 3.34, *p* = 0.04. This is not statistically significant after adjusting the *p*-value to 0.025 (Bonferroni correction). In the Romanian sample participants in the terrorism condition reported higher terrorism threat perception (*M* = 2.49, SD = 0.71) than participants in the crisis (*M* = 2.14, SD = 0.77) and control conditions (*M* = 2.18, SD = 0.68), *F*(2,141) = 3.18, *p* = 0.04. Additionally, participants in the crisis condition reported lower crisis threat perception (*M* = 2.44, SD = 0.86) than participants in the terrorism (*M* = 2.86, SD = 0.77) and control condition (*M* = 2.70, SD = 0.72), *F*(2,141) = 3.44, *p* = 0.04. This is not statistically significant after adjusting the *p*-value to 0.025 (Bonferroni correction) and (in the case of crisis threat) also theory inconsistent. We cannot reject the null hypothesis that the manipulation did not provoke any change in the participants’ threat perceptions and attitudes toward the EU, therefore we collapsed the independent groups and conducted OLS regression analyses controlling for the experimental treatments.

In the first block, we include gender, parents’ education, national identity, political ideology, and European identity. In the second block we include the manipulations (terrorism and economic threat manipulations), and in the last block we included the three measures of threat (economic, immigration and terrorism threats) to control for their sporadic effects.

The models illustrated in Table 3 show that immigration threat is the most important predictor of support for Eurosceptic parties. The perceived threat of terrorism is positively associated with support for Eurosceptic parties in the United Kingdom and Romania, but not in Italy and France. Economic threat is not associated with support for Eurosceptic parties. The models in Table 4 show that perceived terrorism threat was positively associated with Euroscepticism in the United Kingdom and Romanian samples, and immigration threat is associated with support for Euroscepticism in the United Kingdom and Italian samples.

**TABLE 3:** Standardized regression coefficients on the support for Eurosceptic parties (higher values mean higher support for Eurosceptic parties).

	**United Kingdom (*N* = 197)**	**France (*N* = 164)**	**Italy (*N* = 312)**	**Romania (*N* = 144)**	**Full sample (*N* = 817)**
Gender (female)	0.02	0.05	–0.05	0.06	0.06
Parents’ education	–0.03	−0.20^*^	–0.03	–0.06	–0.14^∗∗∗^
National pride	0.26^∗∗∗^	0.23^∗∗^	0.00	0.21^*^	0.10^∗∗^
Political ideology	0.21^∗∗^	0.22^∗∗^	0.50^∗∗∗^	0.13	0.35^∗∗∗^
EU identity	–0.24^∗∗^	–0.10	–0.20^∗∗∗^	–0.02	–0.09^∗∗^
Block 1 Adjusted R^2^	0.18	0.12	0.28	0.03	0.17
Gender (female)	0.02	0.05	–0.04	0.07	0.06
Parents’ education	–0.03	−0.20^*^	–0.02	–0.06	–0.14^∗∗∗^
National pride	0.26^∗∗∗^	0.23^∗∗^	0.00	0.21^*^	0.10^∗∗^
Political ideology	0.21^∗∗^	0.22^∗∗^	0.50^∗∗∗^	0.11	0.35^∗∗∗^
EU identity	–0.23^∗∗^	–0.11	–0.20^∗∗∗^	–0.03	–0.09^∗∗^
Crisis manipulation	0.04	–0.04	–0.03	−0.19^*^	–0.06
ISIS manipulation	–0.02	–0.02	–0.08	0.03	–0.06
Block 2 Adjusted R^2^	0.17	0.11	0.28	0.06	0.17
Gender (female)	–0.04	–0.02	−0.10^*^	–0.01	–0.02
Parents’ education	–0.02	–0.21^∗∗^	0.00	–0.06	−0.07^*^
National pride	0.18^∗∗^	0.18^*^	0.00	0.22^∗∗^	0.10^∗∗^
Political ideology	0.10	0.03	0.31^∗∗∗^	0.08	0.23^∗∗^
EU identity	−0.16^*^	–0.10	–0.16^∗∗^	–0.08	–0.09^∗∗^
Crisis manipulation	0.00	–0.04	–0.04	–0.17	–0.03
ISIS manipulation	–0.04	0.02	–0.08	–0.03	–0.03
Economic threat	0.03	0.07	0.09	0.05	0.14^∗∗∗^
Immigration threat	0.29^∗∗∗^	0.44^∗∗^	0.37^∗∗^	0.11	0.31^∗∗∗^
Terrorism threat	0.23^∗∗^	0.03	0.09	0.29^∗∗^	0.13^∗∗∗^
Block 3 Adjusted R^2^	0.33	0.24	0.41	0.14	0.326
Block 3 F-value	10.00	6.09	21.00	3.14	0.38.66

^*^p < 0.05, ^∗∗^*p* < 0.01, ^∗∗∗^*p* < 0.00.

**TABLE 4:** Standardized regression coefficients on the support for Euroscepticism (higher values mean more Euroscepticism).

	**United Kingdom (*N* = 197)**	**France (*N* = 164)**	**Italy (*N* = 312)**	**Romania (*N* = 144)**	**Full sample (*N* = 817)**
Gender (female)	–0.04	–0.04	–0.10	0.09	–0.02
Parents’ education	–0.10	0.00	–0.08	0.13	–0.12^∗∗∗^
National pride	0.21^∗∗^	–0.03	0.12^*^	0.26^∗∗^	0.15^∗∗∗^
Political ideology	0.13	0.16	0.20^∗∗∗^	0.07	0.16^∗∗∗^
EU identity	–0.31^∗∗∗^	−0.17^*^	–0.43^∗∗∗^	–0.40^∗∗∗^	–0.32^∗∗∗^
Block 1 Adjusted R^2^	0.17	0.03	0.24	0.19	0.15
Gender (female)	–0.05	–0.04	–0.09	0.09	–0.02
Parents’ education	–0.10	0.00	–0.08	0.13	–0.12^∗∗∗^
National pride	0.20^∗∗^	–0.04	0.12^*^	0.26^∗∗^	0.15^∗∗∗^
Political ideology	0.13	0.16	0.20^∗∗∗^	0.06	0.16^∗∗∗^
EU identity	–0.30^∗∗∗^	–0.17	–0.42^∗∗∗^	–0.40^∗∗∗^	–0.32^∗∗∗^
Crisis manipulation	0.03	0.02	0.01	–0.09	–0.01
ISIS manipulation	–0.06	–0.00	–0.05	0.06	–0.02
Block 2 Adjusted R^2^	0.17	0.02	0.24	0.19	0.15
Gender (female)	–0.10	–0.07	–0.09	0.02	–0.05
Parents’ education	–0.90	–0.00	–0.05	–0.12	–0.09^∗∗^
National pride	0.15^*^	–0.07	0.12^*^	0.29^∗∗∗^	0.15^∗∗∗^
Political ideology	0.05	0.08	0.08	0.04	0.10^∗∗^
EU identity	–0.27^∗∗∗^	–0.17	–0.38^∗∗∗^	–0.46^∗∗∗^	–0.31^∗∗∗^
Crisis manipulation	0.01	0.02	0.02	–0.06	0.00
ISIS manipulation	–0.07	0.01	–0.04	0.02	–0.01
Economic threat	–0.02	–0.30	0.04	0.10	0.05
Immigration threat	0.17^*^	0.17	0.28^∗∗∗^	0.02	0.16^∗∗∗^
Terrorism threat	0.20^*^	0.09	–0.05	0.20^*^	0.03
Block 3 Adjusted R^2^	0.23	0.03	0.28	0.24	0.18
Block 3 F-value	6.45	1.55	12.58	5.22	18.02

^*^p < 0.05, ^∗∗^*p* < 0.01, ^∗∗∗^*p* < 0.001.

### Discussion

Making terrorism and economic threats salient did not, in itself, increase support for Euroscepticism in any of the countries. In fact, the only significant effect from either manipulation was that support for Eurosceptic political parties was reduced in Romania by making the economic crisis more noticeable. This effect can be explained by the fact that the EU is seen as having a protective role in the event of economic threat in the Romanian sample. A *post hoc* power analysis revealed that a sample of approximately 118 would be needed to obtain statistical power at the recommended 0.80 level (Cohen, 1988) to detect an effect of 0.25 size. We are therefore confident that our sample size of 197 people (France), 164 (United Kingdom), 312 (Italy), and 144 (Romania) was large enough to detect the effect of the manipulations.

These zero effects were not anticipated, but they are noteworthy nevertheless. Given the unique socio-political climate at the time of data collection (i.e., heightened concerns about terrorism in principal but also about the economic crisis in Europe, especially in France where the data was collected a few months after the Charlie Hebdo attacks, which could have led to concerns about political efforts to exploit terrorism fears), it is possible that our samples were particularly resistant to political manipulation. Regardless, from a moral point of view, given increasing concerns about the political use of “push polling” (i.e., priming reactions by asking specific questions about candidates and policies) raised in relation to the United Kingdom referendum to leave the EU, these zero findings are reassuring because they show that European citizens are resistant to such manipulations (Crick, 2016).

Stronger perceptions of terrorism threat were associated with higher support for Eurosceptic parties, but not in all countries. Specifically, we found that perceived terrorism threat predicted increased support for Eurosceptic parties and negative attitudes to the EU in the United Kingdom and Romania, but not in France and Italy.

## General Discussion

The key aim of this article was to investigate whether perceived terrorism threat contributes to models explaining support for Eurosceptic parties and Euroscepticism above and beyond other relevant variables that have been explored in previous research. Our analyses provided limited support for this hypothesis. Judging by the size of the regression coefficients in both Studies 1 and 2, terrorism threat is a smaller predictor of Euroscepticism than the perceived threat of immigration. The small effect of terrorism threat is present over and above the effect of other threats that are well-established predictors of out-group attitudes, such as immigration and economic threats. Yet, this effect is not consistently found across country samples.

This finding adds to the current literature about the role of perceived threat in explaining political attitudes. The perceived threat of immigration, which encompasses both realistic and symbolic threats, has a larger effect on attitudes to the EU than the perceived threat of terrorism, which confirms previous literature on the topic (McLaren and Johnson, 2007; Pereira et al., 2009; Uenal, 2016). In this article we find that terrorism threat has a small effect on political attitudes over and above the effect of other threats, possibly because of its impact on perceptions of personal safety (Oswald, 2005) and on existential concerns (Pyszczynski et al., 2003), which the threat of terrorism makes more salient than the threats of immigration and economic downturn. However, we find that the effect is not consistent across countries. The reason why the effect of terrorism perceived threat is not consistently found across EU country contexts is that both terrorism and the EU are complex and multifaceted constructs that relate to in-group and out-group identities in different ways. For example, in not all country contexts Euroscepticism and support for Eurosceptic parties are consistent with policy choices such as restrictions on immigration, strict border control, increased surveillance of religious minorities (especially Muslim minorities), which previous studies found to be associated with higher perception of terrorism threat (see for example Huddy et al., 2005; Uenal, 2016; Vergani and Tacchi, 2016). Moreover, terrorism can be perceived both as threat coming from outside the EU (for example, from Middle East or Africa), and as a threat originating from second generations of migrants who are EU citizens, too. In some contexts the perception of terrorism threat might trigger EU identity, and in others national identity, depending on how terrorism is constructed in the media and national discourse. This complex definition of identities in relation to the perceived threat of terrorism and the EU is likely to diversify the effect of terrorism perceived threat on Euroscepticism to the extent of making it, on average across the EU, small or negligible, but significant within many country contexts.

Future research should test whether policy preferences such as support for tougher immigration policies mediate the relationship between the perception of terrorism threat and support for Euroscepticism and Eurosceptic parties. If this mediating relationship exists, it would explain why we see varying results across different countries, as shown in Study 1, where the relationship between terrorism threat and attitudes toward the EU can be positive, negative, or non-significant, and in Study 2, where a significant positive relationship between perceived terrorism threat and Euroscepticism is present in the United Kingdom and Romania, but not in Italy and France. It is also possible that in Italy and in Romania, where there was a relatively higher support for the EU than in other comparable EU countries in 2014 and 2015, many citizens saw the EU as a protective shield from external threats such as economic crisis and terrorism. In the same countries, the majority of citizens with conservative ideology were likely to be voters of pro-EU center right parties in 2014 and 2015. This could explain why in Study 1 higher terrorism threat perception is associated with less negative attitudes to the EU. Future research should test this hypothesis.

One limitation is that the relationship between threat perception and support for Euroscepticism that we found in the representative Eurobarometer sample might be different across age groups, which could explain why it might not be present in the country samples of university students used in Study 2. This is another hypothesis that should be studied in *ad hoc* studies with larger samples and more nuanced measures of threat.

We propose that terrorism threat should be studied alongside other sources of threat in comparative cross-nation research to understand the causes of important political processes, such as the Brexit vote in June 2016. A take-home message from this research is that future studies of perceived terrorism threat should be contextualized in specific national and cultural settings. According to a socio-functional approach to threat (Cottrell and Neuberg, 2005), terrorism threat will provoke different emotional reactions that vary across groups, depending on the stereotypical knowledge relevant to terrorism in a particular environment. For example, terrorism could provoke a fear reaction and therefore a desire to escape, in contrast to an anger response that would result in a desire for aggression (Huddy et al., 2005).

The current study is the first to test the effects of perceived terrorism threat on the support for Euroscepticism and Eurosceptic parties with a comparative approach, using both representative survey data (Eurobarometer) and convenience samples from selected countries. However, it has limitations because of the limited size of the samples in each country, which does not allow us to test specific hypotheses on subgroups, especially in the experimental studies. Moreover, the questions about terrorism in the Eurobarometer survey do not allow differentiating between components of the perception of terrorism threat, such as personal versus national threat, which have been shown to produce different policy responses (Huddy et al., 2005). In our studies, we decided to use more contextual measures of perceived threat, that is, the threat of immigration, terrorism, and economic downfall. Terrorism and economic downfall threaten people’s established way of life and culture, which relates to symbolic aspects. All of these threats are likely to capture, at least in part, symbolic and material dimensions of threat.

Based on these findings, we suggest that terrorism threat deserves to be further investigated alongside other threats that have been so far considered in the literature, that is, perceived threats brought about by immigration and economic hardship. In conclusion, our research demonstrates that the terrorism threat should not be overlooked in research in the quest to understand factors that drive Eurosceptic attitudes. In addition, its relationship with political attitudes seems to be less linear than might first be thought; therefore, future research needs to be conducted to determine the contextual factors that cause the terrorism threat, which results in different attitudinal and behavioral responses.

## Ethics Statement

This study was approved by the Deakin University Ethics Committee and Monash University Ethics Committee.

## Author Contributions

MV contributed to the design, analysis, and writing of the manuscript, and conducted the data collection. A-MB contributed to the design and writing of the manuscript. AS, CB, and DM contributed to the design and data collection. CM contributed to the design and writing of the manuscript.

## Conflict of Interest Statement

The authors declare that the research was conducted in the absence of any commercial or financial relationships that could be construed as a potential conflict of interest.

## Footnotes

1 The four items used to assess economic and terrorism threat are: “How concerned are you that there will be a terrorist attack/economic crisis on [participant country] soil in the near future?” and “How threatened do you personally feel from the threat of terrorism/economic crisis in [participant country]?”
