# Supplementary material for: United in Diversity, Divided in Adversity? Support for Right-Wing Eurosceptic Parties in the Face of Threat Differs Across Nations
Source: Front Psychol. 2019 Aug 14;10:1880. doi: 10.3389/fpsyg.2019.01880 (PMC6702969; doi:10.3389/fpsyg.2019.01880)
Supplement: Supplementary file 1 [file Data_Sheet_1.docx]

**Supplementary materials**

Partial bivariate correlations, controlling for the experimental manipulations (UK, N=197, below the diagonal and France, N = 164, above the diagonal).

|  | 1 | 2 | 3 | 4 | 5 | 6 | 7 | 8 | 9 | 10 |
| --- | --- | --- | --- | --- | --- | --- | --- | --- | --- | --- |
| 1 Gender (female) | - | -.05 | .02 | .11 | .15 | .13 | .18** | .23** | .08 | -.05 |
| 2 Parents’ education | .05 | - | .19* | .24** | -.01 | -.30** | .21* | -.20* | -.10 | .03 |
| 3 National pride | .05 | .08 | - | .27** | .14 | -.06 | .25** | .10 | .24** | -.02 |
| 4 Political ideology | -.07 | -.02 | .21** | - | -.18* | -.28** | .54** | -.06 | .26** | .17* |
| 5 EU identity | -.05 | .20** | .08 | -.17* | - | .36** | -.12 | .33** | -.10 | -.20* |
| 6 Economic threat | .07 | -.04 | -.01 | -.18* | -.09 | - | -.14 | .50** | .03 | -.09 |
| 7 Immigration threat | -.04 | -.02 | .17* | .40** | -.23** | .03 | - | .23** | .46** | .22** |
| 8 Terrorism threat | .30** | -.04 | .22** | .09 | -.06 | .36** | .33** | - | .18* | .03 |
| 9 Eurosceptic parties | .03 | -.06 | .28** | .30** | -.25** | .11 | .48** | .38** | - | .32** |
| 10 Euroscepticism | -.03 | -.15* | .20** | .22** | -.33** | .06 | .35** | .27** | .61** | - |

Partial bivariate correlations, controlling for the experimental manipulations (Italy, N = 312, below the diagonal and Romania, N = 144, above the diagonal)

|  | 1 | 2 | 3 | 4 | 5 | 6 | 7 | 8 | 9 | 10 |
| --- | --- | --- | --- | --- | --- | --- | --- | --- | --- | --- |
| 1 Gender (female) | - | .05 | -.04 | .14 | -.01 | .21* | .05 | .25** | .08 | .08 |
| 2 Parents’ education | -.14* | - | -.13 | -.02 | .06 | -.00 | .06 | .01 | -.09 | -.19* |
| 3 National pride | -.07 | .02 | - | -.09 | .18* | -.11 | .01 | -.03 | .20* | .20* |
| 4 Political ideology | .12* | .04 | .29** | - | .23** | .05 | -.01 | .20* | .10 | -.04 |
| 5 EU identity | -.05 | .18** | .22** | -.06 | - | .17* | -.20* | .26** | .03 | -.35** |
| 6 Economic threat | .14* | -.18** | -.10 | -.10 | -.02 | - | -.19* | .49** | .13 | .09 |
| 7 Immigration threat | .12* | -.07 | .14* | .50** | -.18** | .03 | - | -.05 | .10 | .07 |
| 8 Terrorism threat | .33** | .09 | .00 | .18** | .09 | .41** | .16** | - | .30** | .14 |
| 9 Eurosceptic parties | .03 | -.03 | .11 | .50** | -.23** | .09 | .56** | .20** | - | .26 ** |
| 10 Euroscepticism | -.04 | -.13* | .09 | .24** | -.41** | .02 | .38** | -.04 | .51** | - |


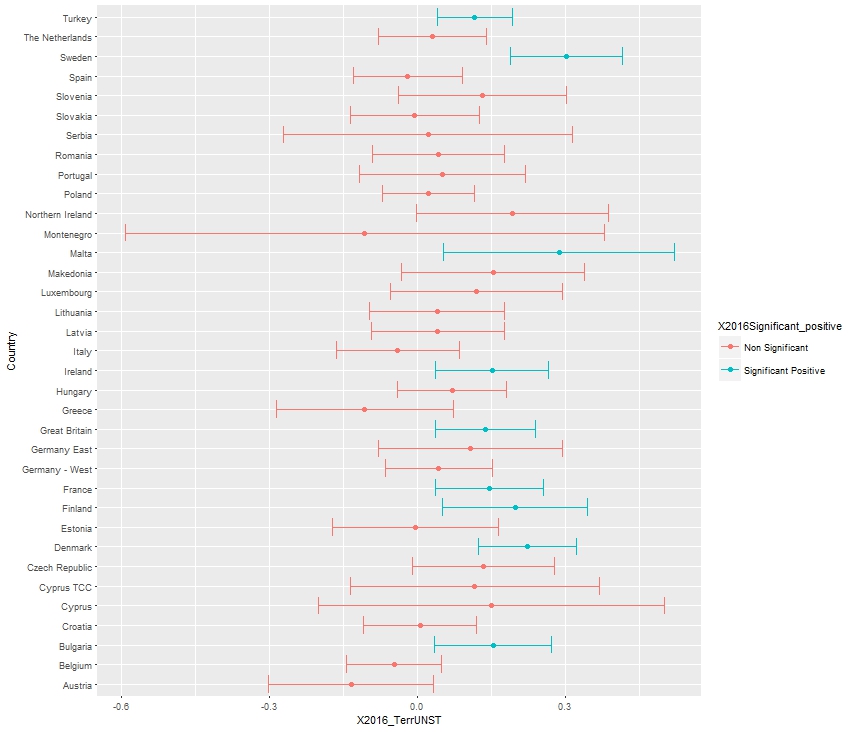


Unstandardized B and Confidence Intervals of the terrorism threat coefficient predicting negative attitudes to the EU, controlling for age, gender, political orientation, realistic and symbolic threats. Data: Eurobarometer 2016.
